# A regulatory role for CD72 expression on B cells and increased soluble CD72 in primary Sjogren’s syndrome

**DOI:** 10.1186/s12865-020-00351-2

**Published:** 2020-04-19

**Authors:** Yuqi Shen, Yuhua Ma, Jingyuan Xie, Li Lin, Yifan Shi, Xiao Li, Pingyan Shen, Xiaoxia Pan, Hong Ren

**Affiliations:** 1 Department of Nephrology, Shanghai Ruijin Hospital, Shanghai Jiao Tong University School of Medicine, 197 Ruijin Er Road, Shanghai, 200025 China; 2 Department of Nephrology, Traditional Chinese Medicine Hospital of KunShan, Suzhou, China

**Keywords:** Primary Sjogren’s syndrome, CD72 expression on B cell, Serum soluble CD72 level, Serum IgG level

## Abstract

**Background:**

CD72, a co-receptor of B cell receptor (BCR), has been reported to have both positive and negative effects on B cell functions in several immunological diseases. The B cell plays an important role in the pathogenesis of primary Sjogren’s syndrome (pSS). However, whether CD72 is involved in the process remains unknown. This study aimed to observe the possible role of CD72 in the pathogenesis of pSS.

**Results:**

A total of 60 cases who fulfilled the American-European Consensus Group (AECG) criteria for the diagnosis of pSS and 61 gender and age-matched healthy controls were recruited in this study. The percentage of CD72+ B cells was 85.31 ± 8.37% in pSS patients and 76.91 ± 8.50% in healthy controls(*p* < 0.001). The percentage of CD72+ B cells was correlated to serum IgG levels in patients [β = 0.018(0.001–0.036), *p* = 0.034]. The level of serum soluble CD72 was significantly higher in pSS patients than the one in healthy controls (0.41 (0.29) vs 0.07 (0.08) ng/mL, *p* < 0.001).

**Conclusions:**

The percentage of CD72+ B cells was upregulated in pSS patients and was correlated to the serum IgG level, which revealed the hyperactivity of B cells in this disease. The serum soluble CD72 level was also increased in pSS patients. These results indicated a potential role of CD72 in the pathogenesis of pSS.

## Background

Primary Sjogren’s syndrome (pSS) is a systemic autoimmune epithelitis characterized by heterogenous clinical manifestations, ranging from xerostomia and xerophthalmia to non-Hodgkin’s lymphoma (NHL) [1, 2]. It affects 0.01–0.1% of the population and has a major impact on patients’ quality of life [3–5]. B cell hyperactivity, reflected by increased serum levels of IgG, IgM, rheumatoid factor (RF) and the presence of anti-SSA and anti-SSB autoantibodies, is a common finding in SS. Hence, B cells play an important role in the pathogenesis of primary Sjogren’s syndrome (pSS) [6].

CD72 is a coreceptor of B cell receptor (BCR), which is expressed on all B cells starting at the pre-B cell stage, but its expression decreases significantly during differentiation into plasma cells. It has a conserved intracellular domain, which contains an immunoreceptor tyrosine-based inhibitory motif (ITIM) that binds SHP-1, a tyrosine phosphatase, and an ITIM-like motif that binds Grb2, an adaptor protein required to activate the Ras pathway [7]. The association between SHP-1 and CD72 forms the basis of a negative signaling function for CD72 [8, 9]. The complex formed by CD72 and Grb2 is a major positive regulator of BCR signaling [10, 11]. CD100, constitutively expressed on T cells, is the natural ligand of CD72 [12, 13]. CD72 is known to play an important role in various B cell processes, including proliferation, apoptosis and differentiation. CD40-induced B cell proliferation was enhanced by ligation of CD72 with CD100, and antigen-mediated B cells proliferation was also enhanced by CD72 ligation [12, 14]. However, other studies revealed that B cells from CD72-deficient mice exhibited increased B cell proliferation in response to BCR ligation [15, 16]. Similarly, CD72 was found to play opposing effects on B cell survival in different studies [8, 16–18]. As for its differentiation, CD72 positively regulates the transition of pre-B cells to mature B cells, and has a mild negative effect on B-1 cell development [15, 19]. Hence, CD72 has both positive and negative effects on B cell functions.

CD72 has been studied in several immunological diseases. The expression of CD72 on B cells decreased while the serum soluble CD72(sCD72) level increased in SLE patients as compared to health controls [20, 21]. Moreover, the expression of CD72 on B cells also decreased in patients with multiple sclerosis [22, 23]. In contrast, the expression of CD72 on B cells was upregulated in pSS patient in the study by Smith et al. [24]. However, the study only included 15 pSS patients, and the expression of soluble CD72 in pSS patients has not been explored till date.

In this study, we investigated the expression of CD72 on B cells as well as the serum soluble CD72 level in pSS patients, and explored whether the CD72 expression is correlated to patients’ clinical features, in order to identify the potential function of this molecular in the pathogenesis of primary Sjogren’s syndrome.

## Results

### CD72 expression on B cells

**Fig. 1:**
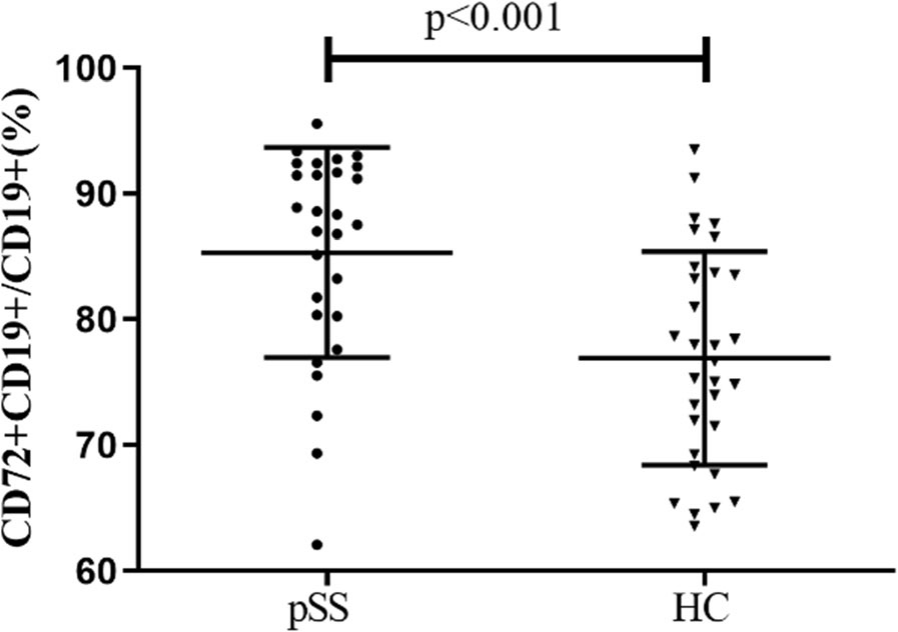
CD72 expression on CD19(+) B cells. pSS, primary Sjogren’s syndrome; HC: healthy control

According to our results, the mean fluorescence intensity (MFI) of CD72 on CD19+ B cells was slightly but not statistically higher in pSS patients compared to that in healthy controls (11,421.86 ± 3725.03 vs 10,350.81 ± 1451.95, *p* = 0.14). However, the percentage of CD72+ B cells was significantly increased in patients than in controls (85.31 ± 8.37% vs 76.91 ± 8.50%, *p* < 0.001) (Fig. 1).

### Association between CD72 expression on B cells and serum indicators

**Fig. 2:**
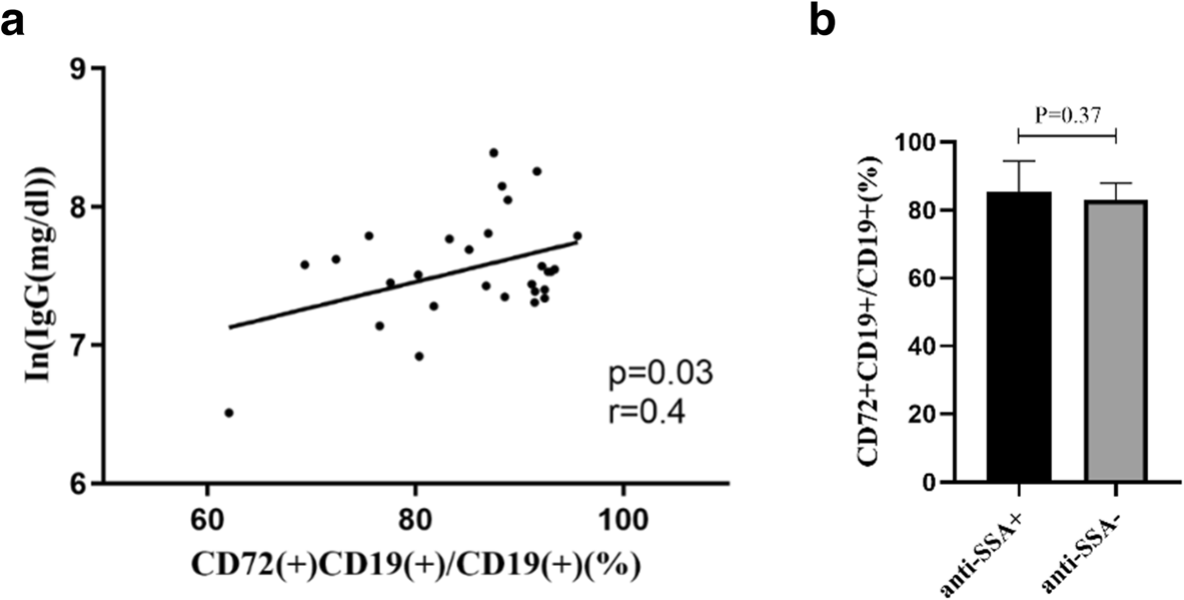
**a** Correlations between CD72 expression on CD19(+) B cells and serum IgG level; **b** CD72 expression on CD19(+) B cells from patients with positive or negative anti-SSA antibodies

The result of Pearson’s correlation coefficient showed that the percentage of CD72+ B cells was positively correlated with the serum IgG level (*p* = 0.03, r = 0.4) (Fig. 2a). The correlation persisted after adjusting for age and gender with linear regression analyses (backward) [β = 0.018(0.001–0.036), *p* = 0.034]. However, a significant difference of the percentage of CD72+ B cells was not found between patients with positive anti-SSA antibodies and those with negative antibodies (Fig. 2b).

### Expression of serum CD72

**Fig. 3:**
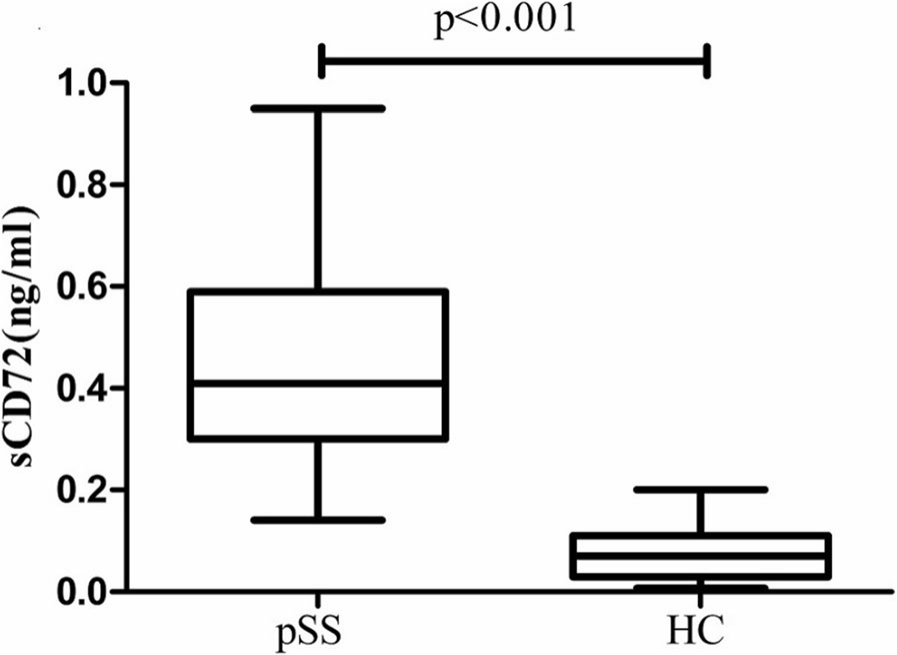
A boxplot of expression of serum soluble CD72 from pSS patients or healthy controls. sCD72: soluble CD72; pSS, primary Sjogren’s syndrome; HC: healthy control

**Fig. 4:**
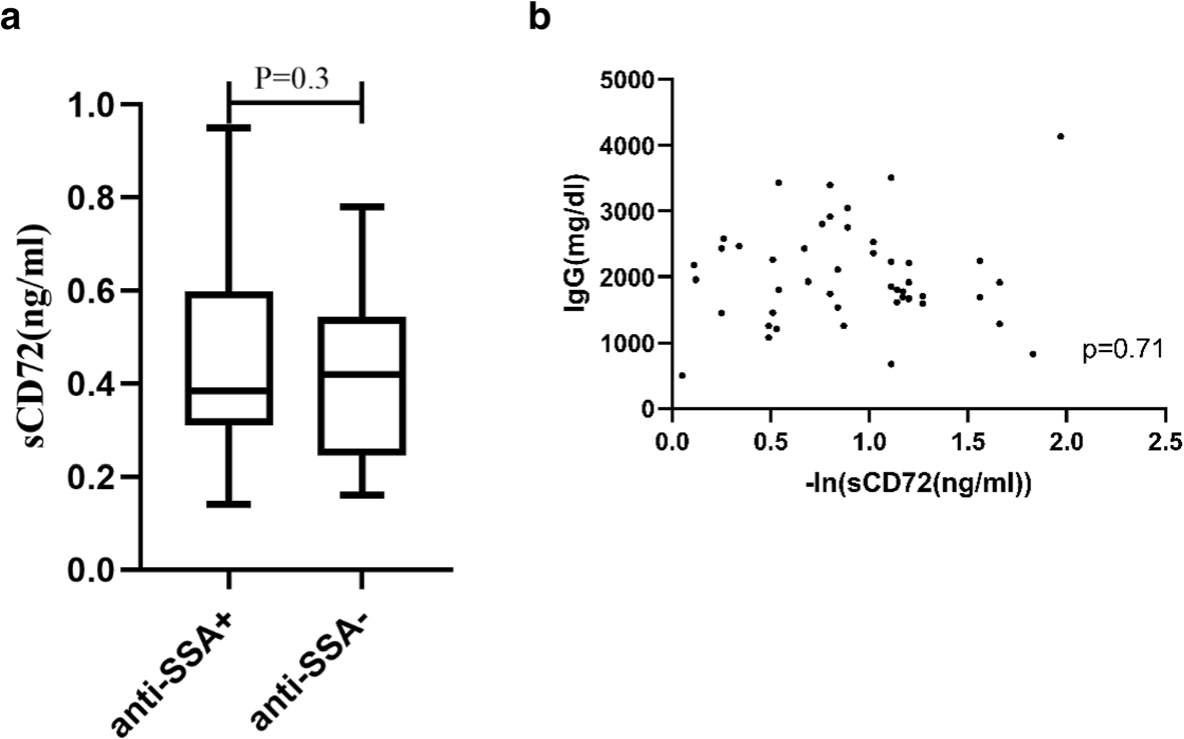
**a** The serum sCD72 levels from patients with positive or negative anti-SSA antibodies; **b** Correlations between the serum sCD72 levels and the serum IgG levels

As shown in Fig. 3, the serum soluble CD72 level was significantly higher in pSS patients than in healthy controls [0.41(0.29) vs 0.07(0.08) ng/mL, *p* < 0.001]. However, there was no significant correlations between the sCD72 levels and the serum levels of anti-SSA antibodies [anti-SSA+ vs anti-SSA-: 0.39 (0.29) ng/mL vs 0.42 (0.3) ng/mL, *p* = 0.3] as well as IgG (*p* = 0.71) (Fig. 4a and b).

## Discussion

The involvement of B lymphocytes has been increasingly recognized in the occurrence of primary Sjogren’s Syndrome (pSS) [6]. And CD72, being a co-receptor of B cell receptor (BCR), has been reported to be an important regulator in the pathogenesis of several autoimmune diseases [20–23, 25]. However, the role of CD72 in pSS has been rarely studied [24]. Here, we examined the expression of CD72 on B cells, and for the first time, the level of the soluble form of CD72 in pSS patients. Our findings suggest that expression of CD72 is correlated to the hyperactivity of B cells and plays a role in the pathogenesis of pSS.

Several studies have reported the aberrant expression of CD72 on B cells in autoimmune diseases and suggested that it has both positive and negative effects on B cell development and function. In SLE patients, the lower expression of CD72 on B cells was inversely associated with patients’ disease activity (SLEDAI) and correlated with the presence of lupus nephritis, anti-dsDNA antibodies and low levels of complement [20]. In multiple sclerosis, the expression of CD72 decreased along with an increase in expression of its ligand CD100 on T cells and abnormal levels of several inflammatory factors [22, 23]. Conversely, CD72 expression was upregulated on CD19 + CD27+ memory B cells in immune thrombocytopenia (ITP), and was associated with platelet count and anti-platelet antibodies [25]. As a negative regulator of BCR signals, once B cells are stimulated by antigens, CD72 molecules would be recruited by BCR signals to the kinase-rich BCR complex and be phosphorylated. The phosphorylated CD72 recruites SHP-1by its ITIM and the CD72-associated SHP-1, in turn, negatively regulates BCR signals. CD72-bound Grb2 can reduce the strength of the negative signals transmitted by CD72 associated SHP-1. Moreover, it has been proved that ligation of CD72 by CD100 can dissociate SHP-1 from CD72. However, CD72 acts as a positive regulator of B cell activation not only by dissociating SHP-1 from CD72 but also by a BCR-independent pathway through activating signals such as btk or mitogen-activated protein kinases (MAPK) by associating with CD19 [7]. Our data demonstrated that the expression of CD72 on CD19+ B cells was upregulated in pSS patients as compared to healthy controls, which was consistent with the findings of Smith et al. [24]. Furthermore, we found that CD72 expression on B cells was closely correlated with patients’ serum IgG level. These observations suggested that CD72 positively regulates B cell functions in pSS and its expression on CD19+ B cells is associated with the hyperactivity of B cells in pSS patients.

Moreover, we assessed the level of serum soluble CD72 in pSS patients for the first time. A previous study found that sCD72 was higher in SLE patients and its level significantly correlated to SLEDAI, the presence of aCL, dsDNA antibodies and lupus nephritis [21]. Similarly, our data showed that sCD72 also increased in pSS patients. According to Zahava Vadasz, et al., such upregulation of sCD72 might be due to the dysregulation of B cells and more intense shedding of CD72 from the membrane of B cells in response to stimulation. Hence, the increase of sCD72 in patients’ serum indicates the hyperactivity of B cells [21].

## Conclusions

In summary, this study found that the expression of CD72 on CD19+ B cells was upregulated in pSS patients and was closely correlated with patients’ serum IgG levels, which suggests that CD72 positively regulates B cell functions in pSS patients. For the first time, this study found that the serum soluble CD72 was also increased in pSS patients which might be attributed to the hyperactivity of B cells. Future studies should be designed to investigate the specific role of CD72 in the pathogenesis of pSS.

## Methods

### Patients

A total of 60 pSS patients from the Department of Nephrology of Ruijin Hospital and 61 gender and age-matched healthy controls (females: 55 vs 56, *p* = 1; mean age: 46.9 ± 13.16 vs 43.25 ± 11.22 years old, *p* = 0.46) were recruited in this study. All patients fulfilled the American-European Consensus Group (AECG) criteria for the diagnosis of pSS. Specific inclusion criteria are as follows: (1) ocular symptom; (2) oral symptoms; (3) positive antibodies to SSA or SSB antigens, or both; (4) Unstimulated whole salivary flow ≤1.5 ml/15 min; (5) Schirmer’s test ≤5 mm per 5 min; (6) ≥50 inflammatory cells/4 mm2 of Minor labial salivary gland on biopsy. Patients who have the presence of any 4 of 6 above items (either item 3 or 6 was positive) or 3 of the latter four items were diagnosed as pSS. Exclusion criteria include active hepatitis C virus infection on polymerase-chain-reaction assay, acquired immunodeficiency disease, radiotherapy of head and neck, graft versus host disease, receipt of anticholinergic drugs, history of lymphoma, IgG4-related disease, sarcoidosis and another well-defined connective tissue disease.

Salivary gland biopsies were performed in all patients and typical performances of Sjogren’s syndrome were found in 59 (98.33%) samples. The unstimulated whole salivary flow test was assessed in 42 cases, and 40 (95.24%) of which met the criteria for SS. The Schirmer’s test was conducted in 28 cases, and 25 (89.29%) cases fulfilled the criteria for SS. The anti-SSA antibodies were positive in 41 of 60 (68.33%) patients, and the anti-SSB antibodies were positive in 32 (53.33%) patients. The mean serum IgG level, an indicator revealing B cells activity, was 2039.47 ± 753.85 mg/dL in patients (Table 1). Fresh heparinized whole blood samples were available from 28 patients and 31 controls for FCM, while serum sCD72 levels were tested in 47 patients and 30 controls. No patient received any immunosuppressive therapy before blood samples were drawn.

**Table 1:** Characteristics of pSS patients and healthy controls

	pSS	control	*p* value
n	60	61	
Female (n)	55	56	1
Age (Y)	46.9 ± 13.16	43.25 ± 11.22	0.46
Anti-SSA antibodies+ (n (%))	41 (68.33)		
Anti-SSB antibodies+ (n (%))	32 (53.33)		
IgG (mg/dl)	2039.47 ± 753.85		
Unstimulated whole salivary flow≤1.5 ml/15 min (n (%))	40 (95.24)		
Schirmer’s test ≤5 mm per 5 min (n (%))	25 (89.29%)		
≥ 50 inflammatory cells/4 mm2 of Minor labial salivary gland on biopsy (n (%))	59 (98.33)		

*pSS* Primary Sjogren’s syndrome, *IgG* Immunoglobulin G

This study was approved by the Ruijin Hospital Ethics Committee at the Shanghai Jiao Tong University School of Medicine (2010 No. 29) in agreement with the Declaration of Helsinki. All subjects provided written informed consent.

### Flow cytometry (FCM)

**Fig. 5:**
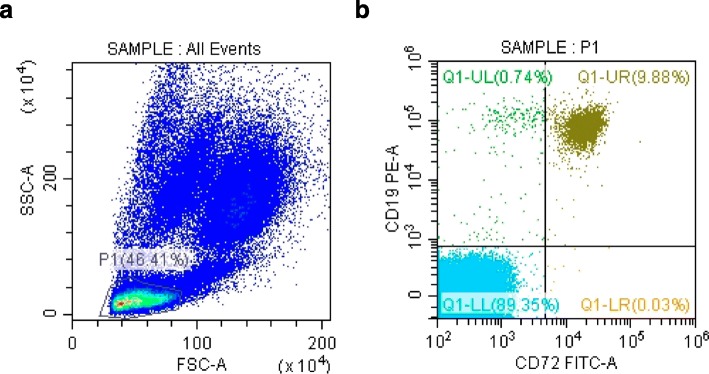
Representative presentation of Flow Cytometer test. **a** FSC-SSC; **b** CD72 FITC-CD19 PE. FSC: forward scatter; SSC: side scatter

20 μL human anti-CD19 PE (BD, US) and anti-CD72 FITC (BD, US) antibodies were added to 100 μL fresh heparinized whole blood samples from pSS patients or controls, and incubated for 30 min in room temperature in the dark. Then 4 ml 1× red blood cell (RBC) lysis buffer was added and mixed thoroughly. After incubation for 15 min, the samples were centrifuged at 1500 rpm for 20 min at 4 °C. The supernatant was discarded and the precipitate was washed with phosphate buffered saline (PBS). Then, 200 μL PBS was added and mixed thoroughly with the precipitate, and the samples were analyzed by FACS (Beckman Coulter). The representative presentation of the Flow Cytometer tests was shown in Fig. 5.

### Analysis of soluble CD72

Serum levels of sCD72 were tested by commercial ELISA kits (MBS2023047, MyBiosource, USA), according to the manufacturer’s instructions.

### Statistical analyses

Statistical analyses were performed with IBM SPSS Statistics version 21.0. Normally distributed, non-normally distributed, and categorical variables were presented as the mean ± SD, median (interquartile ranges, IQRs), or frequency (percentage), respectively. For comparing variables between different groups, t-test and non-parametric tests were used according to the distributional features of the data.

Pearson’s correlation coefficient and linear regression analyses were conducted to detect associations between CD72 expression and clinical parameters.

## Data Availability

The datasets used and/or analyzed during the current study available from the corresponding author on reasonable request.

## Abbreviations

BCR: B cell receptor
pSS: Primary Sjogren’s syndrome
AECG: American-European Consensus Group
IgG: Immunoglobulin G
NHL: Non-Hodgkin’s lymphoma
IgM: Immunoglobulin M
RF: Rheumatoid factor
ITIM: Tyrosine-based inhibitory motif
FCM: Flow cytometry
ELISA: Enzyme-linked immunosorbent assay
MAPK: Mitogen-activated protein kinases
MFI: Mean fluorescence intensity
RBC: Red blood cell
SLEDAI: Systemic Lupus Erythematosus Disease Activity
